# Characterization of the complete mitochondrial genome of *Corvus corone orientalis*

**DOI:** 10.1080/23802359.2019.1622467

**Published:** 2019-07-10

**Authors:** Jin-Qing Jiang

**Affiliations:** College of Animal Science and Veterinary Medicine, Henan Institute of Science and Technology, Xinxiang, China

**Keywords:** *Corvus corone orientalis*, mitochondrial genome, assembly, phylogeny

## Abstract

In this study, the complete mitochondrial genome of *Corvus corone orientalis* was assembled through next-generation sequencing data. This circular mitochondrial genome of *C. corone orientalis* is 16,947 bp in length and has a base composition of A (30.8%), T (24.7%), C (29.9%), and G (14.5%), demonstrating a bias of higher AT content (55.5%) than GC content (44.5%). The mitochondrial genome contains a typically conserved structure among bird mitogenomes, encoding 13 protein-coding genes (PCGs), 22 transfer RNA genes (tRNA), two ribosomal RNA genes (12S rRNA and 16S rRNA), and a control region (D-loop region). Except ND6, all other PCGs were located on the H-strand. ATP8 gene and ATP6 gene were overlapped by 8 bp. The whole mt genome of *C. corone orientalis* and other Corvoidea mitogenomes (24 species, in total) were used for phylogenetic analysis. The result indicated *C. corone orientalis* has the closest relationship with *Corvus cornix cornix* (NC_024698) and clustered within clade of genus *Corvus*.

In this study, the specimen of Oriental Carrion Crow (*Corvus corone orientalis*) was collected in suburb, Shunyi District, Beijing (40°28′N, 116°51′E) and fixed in absolute ethanol. Genomic DNA was extracted from muscle tissues using QLAGEN DNEasy Extraction Kit following the manufactures instructions. The isolated DNA was stored in the sequencing company (HuiTong Tech, Shenzhen, China). Purified DNA was fragmented and used to construct the sequencing libraries following the instructions of NEBNext^®^ Ultra™ II DNA Library Prep Kit (NEB, BJ, CN). Whole genomic sequencing was performed using the Illumina HiSeq 2500 Sequencing Platform (Illumina, CA, USA). Adapters and low-quality reads were removed using the NGS QC Toolkit (Patel and Jain 2012). Then assembly as implemented using SPAdes 3.11.0 (Bankevich et al. 2012). Circularization of this mt genome was confirmed using MITObim V1.9 (Hahn et al. 2013). The complete sequence was primarily annotated by ORF prediction in Unipro UGENE (Okonechnikov et al. 2012) combined with manual correction. All tRNAs were confirmed using the tRNAscan-SE search server (Lowe and Eddy 1997). Other protein coding genes were verified using BLAST search on the NCBI website (http://blast.ncbi.nlm.nih.gov/), and manual correction for start and stop codons were conducted. This complete mitochondrial genome sequence together with gene annotations were submitted to GenBank under the accession numbers of MK714020.

The complete mitochondrial genome of *C. corone orientalis* was 16,947 bp in length and has a base composition of (30.8%), T (24.7%), C (29.9%), and G (14.5%), demonstrating a bias of higher AT content (55.5%) than GC content (44.5%). The mitochondrial genome contains a typically conserved structure among bird mitogenomes, encoding 13 protein-coding genes (PCGs), 22 transfer RNA genes (tRNA), two ribosomal RNA genes (12S rRNA and 16S rRNA), and a control region (D-loop region). All PCGs were located on the H-strand. ATP8 gene and ATP6 gene were overlapped by 8 bp.

For phylogenetic analysis assessing the relationship of this mitogenome, we selected other 24 Corvoidea mitogenomes and one Muscicapidae (*Niltava davidi*) mt genome to construct genome-wide alignment. The genome-wide alignment of all mt genomes was done by HomBlocks (Bi et al. 2018) under trimAl method, which containing all phylogenetic informative gaps in alignments, resulting in 15,868 positions in total. The whole genome alignment was analyzed by IQ-TREE version 1.6.6 (Nguyen et al. 2015) under the GTR + F+R4 model. The tree topology was verified under 1000 bootstrap. As shown in Figure 1, the phylogenetic positions of these 26 mt genomes were successfully resolved with high bootstrap supports except with nine nodes. The result indicated *C. corone orientalis* has the closest relationship with *C. cornix cornix* (NC_024698) and clustered within clade of genus *Corvus*.

**Figure 1.**
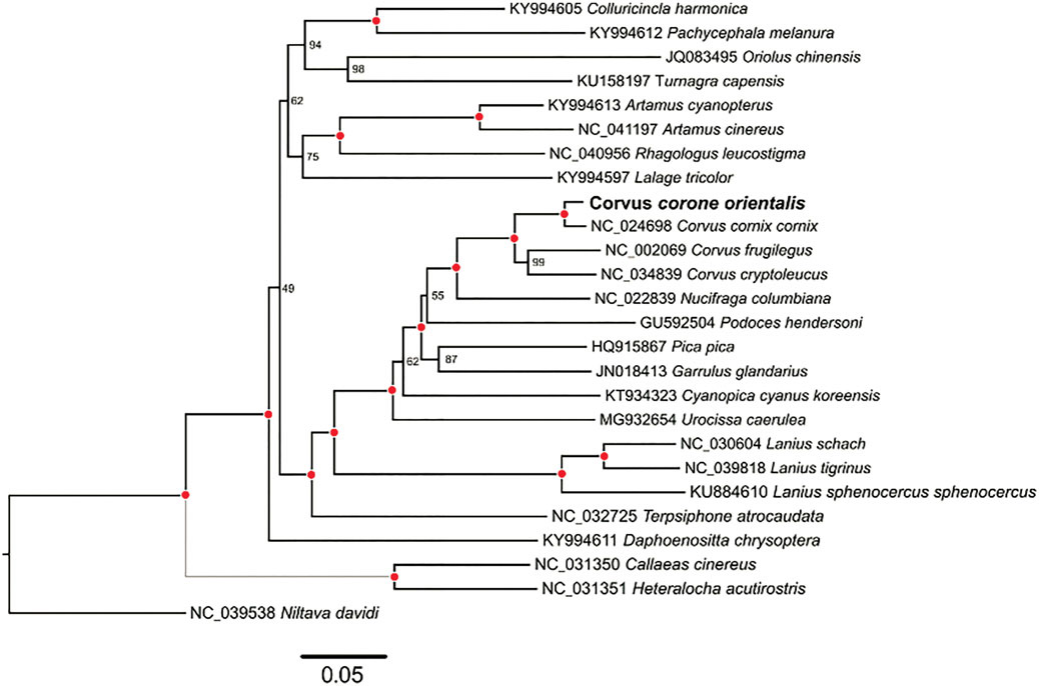
Phylogenetic tree yielded by IQ-TREE of 26 Passeriformes mitogenomes. Consensus tree is shown with support indicated by numbers at branches, representing percentages of bootstraps. Outgroup of the tree was referred to *Niltava davidi*.
